# Supplementary material for: Likelihood of infectious diseases due to lack of exclusive breastfeeding among infants in Bangladesh
Source: PLoS One. 2022 Feb 16;17(2):e0263890. doi: 10.1371/journal.pone.0263890 (PMC8849615; doi:10.1371/journal.pone.0263890)
Supplement: S1 Table — (DOCX) [file pone.0263890.s001.docx]

**S1 Table:** List of variables with their respective definition and value labels

| **Category of variables** | **Variables/description of variables** | **Value Labels** |
| --- | --- | --- |
| Variables on infectious diseases | Had diarrhea recently? | 0 = No & 1= Yes |
|  | Had fever in last two weeks? | 0 = No & 1= Yes |
|  | Had cough in last two weeks? | 0 = No & 1= Yes |
| Variables on Breastfeeding | Currently breastfeeding? | 0 = No & 1= Yes |
| Variables on feeding complementary foods | Gave child sugar water | 0 = No/don’t know & 1= Yes |
|  | Gave child herbal tea | 0 = No/don’t know & 1= Yes |
|  | Gave child fresh milk | 0 = No/don’t know & 1= Yes |
|  | Gave child meat | 0 = No/don’t know & 1= Yes |
|  | Banana, papaya, mango (CS) | 0 = No/don’t know & 1= Yes |
|  | Dal (CS) | 0 = No/don’t know & 1= Yes |
|  | Green leafy vegetables | 0 = No/don’t know & 1= Yes |
|  | Food made from local grain | 0 = No/don’t know & 1= Yes |
|  | Gave child eggs, fish, poultry | 0 = No/don’t know & 1= Yes |
|  | Given in 7 days: Sugar water | 0 = No/don’t know & 1= Yes |
|  | Given in 7 days: Cow's/Goat milk | 0 = No/don’t know & 1= Yes |
|  | Given in 7 days: Other liquids | 0 = No/don’t know & 1= Yes |
|  | Given in 7 days: Banana/Papaya | 0 = No/don’t know & 1= Yes |
|  | Given in 7 days: Green Vegetable | 0 = No/don’t know & 1= Yes |
|  | Given in 7 days: Rice, wheat | 0 = No/don’t know & 1= Yes |
|  | Given in 7 days: Meat/Fish | 0 = No/don’t know & 1= Yes |
|  | Given in 7 days: Dal | 0 = No/don’t know & 1= Yes |
|  | Given in 7 days: Other | 0 = No/don’t know & 1= Yes |
|  | Gave child CS liquid: sugar water/honey/juice | 0 = No/don’t know & 1= Yes |
|  | Gave child CS liquid: cow's or goat's milk or yogurt | 0 = No/don’t know & 1= Yes |
|  | Gave child CS foods: meat/fish/eggs | 0 = No/don’t know & 1= Yes |
|  | Gave child plain water? | 0 = No/don’t know & 1= Yes |
|  | Gave child juice? | 0 = No/don’t know & 1= Yes |
|  | Gave child tinned, powdered or fresh milk? | 0 = No/don’t know & 1= Yes |
|  | Gave child baby formula? | 0 = No/don’t know & 1= Yes |
|  | Gave child fortified baby food (cerelac, etc)? | 0 = No/don’t know & 1= Yes |
|  | Gave child soup/clear broth? | 0 = No/don’t know & 1= Yes |
|  | Gave child other liquid? | 0 = No/don’t know & 1= Yes |
|  | Gave child bread, noodles, other made from grains? | 0 = No/don’t know & 1= Yes |
|  | Gave child potatoes, cassava, or other tubers? | 0 = No/don’t know & 1= Yes |
|  | Gave child eggs? | 0 = No/don’t know & 1= Yes |
|  | Gave child meat (beef, pork, lamb, chicken, etc)? | 0 = No/don’t know & 1= Yes |
|  | Gave child pumpkin, carrots, squash (yellow or orange inside)? | 0 = No/don’t know & 1= Yes |
|  | Gave child any dark green leafy vegetables? | 0 = No/don’t know & 1= Yes |
|  | Gave child mangoes, papayas, other vitamin A fruits? | 0 = No/don’t know & 1= Yes |
|  | Gave child any other fruits? | 0 = No/don’t know & 1= Yes |
|  | Gave child liver, heart, other organs? | 0 = No/don’t know & 1= Yes |
|  | Gave child fish or shellfish? | 0 = No/don’t know & 1= Yes |
|  | Gave child food made from beans, peas, lentils, nuts? | 0 = No/don’t know & 1= Yes |
|  | Gave child cheese, yogurt, other milk products? | 0 = No/don’t know & 1= Yes |
|  | Gave child other solid-semisolid food? | 0 = No/don’t know & 1= Yes |
|  | Gave child yogurt? | 0 = No/don’t know & 1= Yes |
|  | Drank from bottle with nipple? | 0 = No/don’t know & 1= Yes |
|  | Drank from bottle with nipple yesterday/last night? | 0 = No/don’t know & 1= Yes |
|  | Did eat any solid, semi-solid or soft foods yesterday? | 0 = No/don’t know & 1= Yes |
|  | Given child anything other than breast milk? | 0 = No/don’t know & 1= Yes |
| Covariates that may increase the risk of infectious diseases | Type of place of residence | 1 = Urban & 2 = Rural |
|  | Division | 1. Barisal 2. Chittagong 3. Dhaka 4. Khulna 5. Mymensingh 6. Rajshahi 7. Rangpur 8. Sylhet |
|  | Respondent’s highest educational level | 1. No education 2. Primary 3. Secondary 4. Higher |
|  | Source of drinking water | 1. Piped 2. Tubewell 3. River/Pond/Surface/Rain/etc |
|  | Type of toilet facility | 1. Modern 2. Pit latrine 3. Others type latrine 4. No facility |
|  | Main floor material | 1. Katcha 2. Pacca 3. Others |
|  | Husband/partner's education level | 1. No education 2. Primary 3. Secondary 4. Higher |
|  | Husband/partner's occupation | 1. Agriculture 2. Non-agriculture 3. Unskilled 4. Skilled 5. Professional 6. Big business 7. Small business 8. Others |
|  | Sex of child | 1 = Male & 2 = Female |
|  | Received BCG? | 1. Not received BCG 2. Received BCG |
|  | Survey year | 1. 1996-97 2. 1999-00 3. 2003-04 4. 2007 5. 2011 6. 2014 7. 2017-18 |
