# Supplementary material for: Likelihood of infectious diseases due to lack of exclusive breastfeeding among infants in Bangladesh
Source: PLoS One. 2022 Feb 16;17(2):e0263890. doi: 10.1371/journal.pone.0263890 (PMC8849615; doi:10.1371/journal.pone.0263890)
Supplement: S2 Table — (DOCX) [file pone.0263890.s002.docx]

**S2 Table:** Summary of the datasets

| **BDHS survey year** | **Number of cases after discarding the cases with infants aged more than 6 months** | **Final datasets after discarding the cases with missing values** |
| --- | --- | --- |
| 1996-97 | 1299 | 687 |
| 1999-00 | 1347 | 826 |
| 2003-04 | 1331 | 794 |
| 2007 | 1044 | 633 |
| 2011 | 1403 | 928 |
| 2014 | 1150 | 764 |
| 2017-18 | 1559 | 1092 |
| Total | 9133 | 5724 |
