# Supplementary material for: Likelihood of infectious diseases due to lack of exclusive breastfeeding among infants in Bangladesh
Source: PLoS One. 2022 Feb 16;17(2):e0263890. doi: 10.1371/journal.pone.0263890 (PMC8849615; doi:10.1371/journal.pone.0263890)
Supplement: Attachment — Submitted filename: Response to Reviewers.pdf [file pone.0263890.s004.pdf]

Author responses to the review comments:

We would like to express our sincere gratitude to the three reviewers and the Academic Editor for their valuable comments. We have considered all the comments made by the reviewers and thoroughly revised and formatted the manuscript accordingly. A detailed response to each of the comments is provided in the table below:

**Academic Editor comments:**	**Response**	**Note**
Please include the following items when submitting your revised manuscript:<br>• A rebuttal letter that responds to each point raised by the academic editor and reviewer(s). You should upload this letter as a separate file labeled 'Response to Reviewers'.<br>• A marked-up copy of your manuscript that highlights changes made to the original version. You should upload this as a separate file labeled 'Revised Manuscript with Track Changes'.<br>• An unmarked version of your revised paper without tracked changes. You should upload this as a separate file labeled 'Manuscript'.<br><br>If you would like to make changes to your financial disclosure, please include your updated statement in your cover letter. Guidelines for resubmitting your figure files are available below the reviewer comments at the end of this letter.	Thank you very much. The required files are submitted through the submission system.<br><br>We include all required information in the cover letter.	
Journal Requirements:<br><br>When submitting your revision, we need you to address these additional requirements.<br><br>Please ensure that your manuscript meets PLOS ONE's style requirements, including those for file naming. The PLOS ONE style templates can be found at<br><br>https://journals.plos.org/plosone/s/file?id=wjVg/PLOSOne_formatting_sample_main_body.pdf and<br><br>https://journals.plos.org/plosone/s/file?id=ba62/PLOSOne_formatting_sample_title_authors_affiliations.pdf<br><br>2. We suggest you thoroughly copyedit your manuscript for language usage, spelling, and grammar. If you do not know anyone who can	Many thanks. The manuscript is revised according to PLOS ONE's style.<br><br>All necessary files are uploaded in system of the journal.	

<table>
<tr><td>help you do this, you may wish to consider employing a professional scientific editing service.

Upon resubmission, please provide the following:

The name of the colleague or the details of the professional service that edited your manuscript

A copy of your manuscript showing your changes by either highlighting them or using track changes (uploaded as a *supporting information* file)

A clean copy of the edited manuscript (uploaded as the new *manuscript* file).</td><td></td><td></td></tr>
<tr><td>3. We note that you have indicated that data from this study are available upon request. PLOS only allows data to be available upon request if there are legal or ethical restrictions on sharing data publicly. For more information on unacceptable data access restrictions, please see http://journals.plos.org/plosone/s/data-availability#loc-unacceptable-data-access-restrictions.

In your revised cover letter, please address the following prompts:

a) If there are ethical or legal restrictions on sharing a de-identified data set, please explain them in detail (e.g., data contain potentially sensitive information, data are owned by a third-party organization, etc.) and who has imposed them (e.g., an ethics committee). Please also provide contact information for a data access committee, ethics committee, or other institutional body to which data requests may be sent.

b) If there are no restrictions, please upload the minimal anonymized data set necessary to replicate your study findings as either Supporting Information files or to a stable, public repository and provide us with the relevant URLs, DOIs, or accession numbers. For a list of acceptable repositories, please see http://journals.plos.org/plosone/s/data-availability#loc-recommended-repositories.</td><td>Thanks for raising these points.

Data Availability statement:
After registration, the data set is available via the following access link http://dhsprogram.com/data/available-datasets.cfm.

Ethics approval and consent to participate:
Not applicable because this study is based on secondary data analysis. However, the original DHS data collection follows the proper ethical guidelines.

Thanks. We add the statement of ethical approval, data availability, competing interest, funding and acknowledgement after the Conclusion section.

We checked all the references in the reference list and revised them accordingly.

Moreover, we add some references during the revision of the manuscript as per comments.</td><td></td></tr>
</table>

We will update your Data Availability statement on your behalf to reflect the information you provide.<br><br>Please review your reference list to ensure that it is complete and correct. If you have cited papers that have been retracted, please include the rationale for doing so in the manuscript text, or remove these references and replace them with relevant current references. Any changes to the reference list should be mentioned in the rebuttal letter that accompanies your revised manuscript. If you need to cite a retracted article, indicate the article's retracted status in the References list and also include a citation and full reference for the retraction notice.		
**Reviewer 1 comments:**	**Response**	**Note**
Authors have a seemingly well-conducted data analysis of the BDHS. The article is an appropriate one to discuss the role of EBF in the prevention of infectious diseases. The objective is focused and clearly mentioned.<br>The whole manuscript needs to be much more concise throughout. In the overall manuscript, it seems very confusing as Diarrhea, and ARI are not infectious diseases but Infectious diseases (ARI/Diarrhea) are different from the earlier two.<br>There are however some critical methodological and presentation considerations that might improve the manuscript greatly:<br><br>The title might be more precise like, "Likelihood of infectious diseases due to lack of exclusive breastfeeding among infants in Bangladesh".	We highly appreciate this comment.<br><br>Thank you very much. We change the title of the manuscript as per your suggestion.	Revised texts are in red color.<br>Page: 1
Abstract:<br>1. The meaning of the 1st line of the Background section is not clear.<br>2. Line 27: define SDG acronym at first use<br>3. Line 29: EBF, ARI define acronym at first use<br>4. In the result section: only adjusted ORs can be mentioned, authors may omit the crude values. It makes the result section clumsy.<br>5. Conclusion: Line 54 can be like, "Findings of this study emphasize the importance of EBF up to six months of age to prevent diarrhea and ARI………. " and this complex line should be broken down into two simple sentences for better understanding of the reader.<br>6. Define the acronyms: WHO, UNICEF, AAP, AAFP, and NNPE.	Thanks for your in-depth review of the manuscript and potential feedback. We appreciate these comments as they will be helpful to enhance the quality and readability of the manuscript.<br><br>The abstract section is revised accordingly.<br><br>We also add a list of abbreviation after the Conclusion section.	Revised texts are in red color.<br>Page: 2-3 & 20

Introduction:<br>1. Line 80: REB should be elaborated.<br>2. Line 96: can be like "The infants were not exclusively breastfed had a higher likelihood……….<br>3. "Moreover" – the term is used several times like, in line 109, 114…<br>4. Line 119-129: No idea why these pieces of information are in the Introduction section? Repetitive of the abstract.	The authors are grateful to the reviewer for highlighting these points.<br><br>The elaboration of REB is given in the first time use (abstract).<br><br>We change the repetition of the word "Moreover" is revised.<br><br>Line 119-129 are removed from this section and placed in the methodology section.	Revised texts are in red color.<br>Page: 3-5
Materials and Method:<br>1. Conceptual framework: can be replaced by a study flow diagram and "A conceptual framework illustrates the whole sequential procedure of a study." This line can be removed.<br>2. Line 170, 171: currently fed breast-milk….. should be "currently breastfed…..".<br>3. Statistical analysis: Recommend detailing the specific analyses<br>a. Line 199- 209: Logistic Regression Model section is not required here, either author may add a reference.<br>b. during modelling what method was followed in logistic regression analysis (entry, stepwise, etc), not clear?<br>4. It seems like when authors use infectious disease (Diarrhea/ ARI), but only "diarrhea" and only "ARI" are not infectious diseases!! The author needs to rename the variable "infectious disease (Diarrhea/ ARI)".	Thank you very much for pointed out this issue.<br><br>This section is revised as par your guidelines.<br><br>Thanks. We mention the specific model, test and other statistical measures used in this study in this section. The description of the logistic regression model is removed, and we add references here. We also add the name of the method used in logistic regression modelling.<br><br>We appreciate your feedback. According to your suggestion, we have renamed the variable "infectious disease (Diarrhea/ ARI)". We consider diarrhea, ARI separately as well as the occurrence of either diarrhea (D) or ARI or both and named as CoDARI (i.e., combination of D or ARI). We revised the texts.	Revised texts are in red color.<br>Page: 6-9 & 26
Result: Overall the result section is not written in a standard manner, which is not up to the mark for a prestigious journal like PLOS One.<br><br>1. Figure title should be revised. What does it mean by "D, ID, (D/ARI)" should be mentioned.<br>2. Line 232, 235, 236: better to mention exact figure for prevalence like lowest prevalence (), highest prevalence ()…., it is difficult for the author to find out the prevalence from the table/ figure.<br>3. Line 242- 253: why a different p-value was considered, is not clearly mentioned.<br>4. Table 1 only presents p values which is a bit misleading	Thanks for your insightful comments. The manuscript is revised accordingly.<br><br>We mention the meaning of D, ID, (D/ARI) in the revised manuscript and add a list of abbreviations in the last part of the manuscript.<br><br>Thanks. We add the percentage in the bracket.<br><br>Table 1 and texts are revised as per your suggestions (4 and 5).<br><br>We revise the tables as per your guidelines. We replace independent	Revised texts are in red color.<br>Page: 9-16

5. Table 1. Need to mention the comparison group (categories) among the independent variables. Like: type of residence- urban/ rural etc. Better to replace independent variables with "associated factors" and dependent variables with "outcomes"<br>6. Table 1: format should be changed, as it is difficult to understand the p values from 2 rows.<br>7. Line 305: what are the other significant factors which were adjusted?<br>8. Table 2:<br>a. needs to mention what is D, ID, ARI, coefficient beta, SE below the table.<br>b. instead of mentioning p value=0.000, better to use like <0.001.<br>c. better to mention the significance level.<br>9. Line: 325: omit "diseases"	variables with "associated factors" and dependent variables with "outcomes".<br><br>Thanks. We change the format and style of the presentation of Table 1.<br><br>Table 2 is revised, and we mention the meaning of D, ID, ARI, EBF, SE below the table as a note.<br><br>We appreciate your careful checking of this manuscript. We omit "diseases" in Line 325.	
Discussion: Use the discussion to detail how their findings add to the literature. The author just mentioned the similarities of their findings with other literature, but the reason behind those could be highlighted.<br>1. Need to elaborate MICS-2003.<br>2. There is no paragraph found for table 1 in the discussion section. Then why authors look for the significance values of the associated factors is unclear.<br>3. Line: 365-366, 369- 370: better to remove the ORs and 95% CIs from the discussion section.<br>4. Line 375: "not breastfeeding" should be replaced by "non-breastfed infants" are associated…..<br>5. Line 376- 377: omit the RR values.<br>6. It should not be recommended to use too many values in the discussion section.	Thanks for all the insightful comments of the reviewer, which help to improve our research. We have tried to incorporate the valuable comments of the reviewer into the revised manuscript.<br><br>We add the elaboration of MICS.<br><br>We add a section in the discussion part for Table 1.<br><br>Thank you very much. We remove some of the ORs and 95% CIs from the discussion section.<br><br>We delete the RR values and fully agreed with your comments.	Revised texts are in red color.<br>Page: 16-19
Conclusion:<br>1. There is a repetition of some lines from the abstract and Introduction.<br>2. Need to add some lines as a recommendation.	The conclusion section is revised, and some recommendations are added as well.	Revised texts are in red color.<br>Page: 19-20
References:<br>Ref 28: needs to be edited	Thanks for your careful checking. Ref 28 is corrected.	Revised texts are in red color.<br>Page: 25
Others:<br>English and grammar in the manuscript are relatively poor which obscures the readers' understanding throughout much of the work.	he whole manuscript is also edited from an English and grammatical point of view.	Revised texts are in red color.<br>Page: 1-22
**Reviewer 2 comments:**	**Response**	**Note**

Thank you for the opportunity to review this manuscript. It is well written but authors have to work on the following.	Thank you very much for your valuable comment and suggestions that help us improve the manuscript's quality.	
1. Avoid Abbreviation in the title	Thanks. The title is revised as per your comment.	Revised texts are in red color.<br>Page: 1
2. Any abbreviation has to be written in long form in the first time used	We appreciate the feedback. All abbreviation is revised in the texts, and we also present them in the list of abbreviation section.	Revised texts are in red color.<br>Page: 2-3, 8-10, 15, 20
3. The abstract is unnecessarily long, some findings like the chi-square and crude odds ration can be reported in the results section in the main document.	Thanks. The abstract is revised according to your guidelines.	Revised texts are in red color.<br>Page: 2-3
4. In the material and method section a brief description of the conceptual framework is needed before authors refer the reader to the figure.	Thank you very much for carefully checking the manuscript.<br><br>The texts are revised as per your comments.	Revised texts are in red color.<br>Page: 6
5. Table 1 needs to be presented in a more simplified way.	Thanks. We simply present the p-value of the covariates by types of disease over the period considered in this study. Now, we change the style of presentation of Table 1.	Revised texts are in red color.<br>Page: 12-13
6. In table two present the odds ratio and confidence intervals	Many thanks, we appreciate your comment. W present the odds ratio and 95% confidence intervals of the parameters in Fig 3 instead of presenting them all in a Table. This is just another way of presenting info, and we hope you appreciate it..	We present the odds ratio and 95% CI in Figure 3.
7. The discussion has a lot of repetition of the results, interpretation and discussion of results needs to be strengthen	Thank you very much for these points, and we appreciate because it enhances the quality of the manuscript. Therefore, we have revised the discussion section..	Revised texts are in red color.<br>Page: 16-19
**Reviewer 3 comments:**	**Response**	**Note**
Likelihood of Infectious Diseases (Diarrhea/ARI) Due to Lack of Exclusive	Thank you very much for your insightful and impressive comments that will	

Breastfeeding of Infants (0-6 months) in Bangladesh<br><br>This manuscript reports the findings from the quantitative analytical cross-sectional designed study which aimed to measure the likelihood of infectious diseases (diarrhea/ARI) due to lack of Exclusive Breast Feeding (EBF) of infants aged 0-6 months in Bangladesh. The need of this study is demonstrated by the slow reduction of neonatal mortality rate to achieve the SDG-3 and the evidence that most neonatal and infants infectious disease burden are attributed to suboptimal breastfeeding in developing countries.<br><br>This topic is of public health concern in developing countries. There are limited empirical studies in the region so this study has the potential to fill that gap. The strength of this study is the use of large data and the clear description of the method used which may allow the replication of the study.	motivate us to engage with research activities.	
To make this report more meaningful to this journal's readers, authors may have to consider the following comments/ recommendations:<br><br>1. Introduction:<br>• The author refer the reader to see table S1 (line 154, 164 and 168) and table S2 (line 189), but the tables are not included in this submission.<br>• Information in Line 119-127 need to move to methodology section	Thanks for highlighting this issue. We have provided the necessary supplementary files at the time of submission. Maybe there is some reason for missing the files. However, we assure you that we will upload all supporting files when submitting the revised version, including the S1 Table and S2 Table, again.<br><br>Thank. According to your suggestion, we move Line 119-127 to the methodology section.	Revised texts are in red color.<br>Page: 3-5
2. Results:<br>• The demographic characteristics of the study sample need to be described first so as the reader can make a meaningful comparison.<br><br>• It is not clear the difference of information this study need to communicate from Line 297 - 310 and information from line 319 to 331, both are describing the odds of developing infectious diseases (D, ARI, D/ARI) due to lack of EBF if it is not a repetition then this need to be cleared	Thank you very much for these insightful comments.<br><br>The description of the demographic characteristics is added at the beginning of the result section.<br><br>Actually, from Line 297-310, we describe the findings (Table 2) based on the significance of the parameters (p-value). However, from Line 319-331, we present the description of results considering the OR and 95% confidence intervals illustrated in Fig 3.	Revised texts are in red color.<br>Page: 9-16

3. Need to work on grammer and correct use of abbreviations	Thanks. The whole manuscript is revised and edited to correct any grammatical mistakes and typos.<br><br>We add the list of abbreviations in the last section of our manuscript.	Revised texts are in red color.<br>Page: 1-22

Finally, the revised manuscript has been produced following the valuable comments and suggestions of the reviewers. Once again, we would like to thank the reviewers for their sincere dedication, professional insights, and earnest cooperation in reviewing the manuscript.
