# Supplementary material for: Likelihood of infectious diseases due to lack of exclusive breastfeeding among infants in Bangladesh
Source: PLoS One. 2022 Feb 16;17(2):e0263890. doi: 10.1371/journal.pone.0263890 (PMC8849615; doi:10.1371/journal.pone.0263890)
Supplement: Attachment — Submitted filename: plos one review.docx [file pone.0263890.s003.docx]

**Likelihood of Infectious Diseases (Diarrhea/ARI) Due to Lack of Exclusive Breastfeeding of Infants (0-6 months) in Bangladesh**

This manuscript reports the findings from the quantitative analytical cross-sectional designed study which aimed to measure the likelihood of infectious diseases (diarrhea/ARI) due to lack of Exclusive Breast Feeding (EBF) of infants aged 0-6 months in Bangladesh. The need of this study is demonstrated by the slow reduction of neonatal mortality rate to achieve the SDG-3 and the evidence that most neonatal and infants infectious disease burden are attributed to suboptimal breastfeeding in developing countries.

This topic is of public health concern in developing countries. There are limited empirical studies in the region so this study has the potential to fill that gap. The strength of this study is the use of large data and the clear description of the method used which may allow the replication of the study.

To make this report more meaningful to this journal’s readers, authors may have to consider the following comments/ recommendations:

1. Introduction:

- The author refer the reader to see table S1 (line 154, 164 and 168) and table S2 (line 189), but the tables are not included in this submission.
- Information in Line 119-127 need to move to methodology section

2. Results:

- The demographic characteristics of the study sample need to be described first so as the reader can make a meaningful comparison.
- It is not clear the difference of information this study need to communicate from Line 297 -310 and information from line 319 to 331, both are describing the odds of developing infectious diseases (D, ARI, D/ARI) due to lack of EBF if it is not a repetition then this need to be cleared

3. Need to work on grammer and correct use of abbreviations
